# Neural responses to virtual avatars are shaped by user preference and personality traits

**DOI:** 10.1038/s41598-026-39704-z

**Published:** 2026-02-10

**Authors:** Ayumi Takemoto, Motoaki Sugiura

**Affiliations:** 1 Institute of Development, Aging and Cancer, Tohoku University, Sendai, 980-8575 Japan; 2 International Research Institute of Disaster Science, Tohoku University, Sendai, 980-8572 Japan

**Keywords:** Human–computer interaction, Social cognition, Personality traits, Human behaviour, Neuroscience

## Abstract

This study aimed to investigate individual differences’ effects on brain activity in selected and non-selected avatars for re-engagement. The development of some applications for human–computer interaction has accelerated over the past decade. To develop a human–computer communication system using virtual avatars without losing the user’s interest and attention, this study revealed differences in the neural mechanisms underlying the perception of virtual avatars between avatars with which users want to converse again (selected avatars) and those with which they do not (non-selected avatars). Forty-two individuals were recruited; they watched two videos in sequence in which each virtual avatar greeted them, and they then reported which avatars they wanted to talk to again. Meanwhile, brain activities were recorded by functional magnetic resonance imaging (fMRI). After the fMRI recording, the responses to the questionnaires regarding personality traits and avatar impressions were rated. Brain activities were compared along with the score of each personality questionnaire. The results indicated that the left middle temporal gyrus (MTG) was more active in selected compared to non-selected avatars. Furthermore, the brain activities of right superior frontal gyrus (SFG) and the left middle cingulate gyrus (MCG) had a statistically negative correlation with the score of openness in the Ten-Item Personality Inventory (TIPI) in selected avatars. These findings indicate that neural responses during brief avatar evaluation are associated with both avatar selection outcomes and individual personality differences. While these results do not permit direct inferences about specific psychological processes, they provide an insight into the neural correlates of early-stage avatar preference formation in human–computer interaction.

## Introduction

Since the late nineties, applications for human–computer interaction have increasingly incorporated virtual agents and avatars as user-facing interfaces. Such avatars are now widely used in diverse contexts, including information provision, customer support, education, and health-related services. In many of these settings, users could encounter an avatar for the first time and must decide whether to continue interacting with it within a very limited time frame. Previous studies have suggested that the presence and appearance of virtual avatars can influence users’ engagement and evaluative responses^1^. For example, avatar design features such as realism, animacy, and visual customization have been shown to shape users’ preference and willingness to interact with artificial agents^1^.

However, it remains unclear how neural responses differ when individuals evaluate avatars that are selected as opposed to those who are not selected within a brief, first-encounter task, and how such neural responses may be influenced by individual personality traits. To address this gap, this study investigated differential brain activity associated with avatars that were selected versus not selected for re-engagement, based on a task-specific, comparative choice. Importantly, the present study does not aim to model rich or sustained social interactions. Instead, it focuses on a tightly controlled, early evaluation phase in which users make a relative preference decision between two avatars after brief, standardized greeting videos with identical verbal content. This experimental simplification was deliberately adopted to minimize confounding influences of semantic information, conversational dynamics, and task complexity, thereby isolating neural responses associated with appearance-based and immediately perceived social cues.

To examine this issue, 3T functional magnetic resonance imaging (fMRI) was employed to examine neural activity while participants viewed avatar greeting videos and subsequently indicated which avatar they would prefer to interact with again. By comparing brain responses to avatars that were selected as opposed to those not selected for re-engagement with this constrained task context, the present study aims to characterize neural correlates of early preference formation that may not be fully captured by subjective self-report alone. Clarifying these neural patterns may contribute to a more precise understanding of how users evaluate virtual avatars at first contact, providing foundational insights for the design of user-centred avatar-based systems.

In addition to engagement outcomes, prior research has highlighted several factors that influence how users perceive, and respond to, virtual avatars. For instance, Shin, et al., (2019) demonstrated that an avatar’s degree of realism (e.g. hyper-realistic vs. cartoonish) and animacy (e.g. animate vs. still) significantly affects users’ feelings of eeriness, trustworthiness, and decision-making processes^2^. These perceptual features are critical in shaping users’ psychological responses to avatars and, by extension, their willingness to continue engaging with avatar-based systems.

Beyond perceptual realism, demographic characteristics such as race, gender, and age also have been shown to influence users’ evaluation and choice of avatars. In diverse contexts, including marketing, partnership formation and help-seeking, users’ preferences have been shown to be influenced by demographic attributes of avatars^3^. For example, individuals tend to form selection preferences with avatars that share their racial background^4^, and women, in particular, are more likely to report higher affinity to avatars of the same gender in some contexts^5^.

However, these preferences are not uniform across individuals and are often moderated by personality traits. Research ground in the Big-Five personality model^6^ has revealed that traits such as neuroticism, agreeableness, extraversion, and openness predict attitudes toward social agents such as robots and avatars. Dunn, et al., (2012) found that both men and women with greater agreeableness showed a stronger preference for avatars that resemble themselves, while both men and women higher in neuroticism and introversion were more likely to build attractive avatars^7^. Individuals high in neuroticism tend to harbour negative attitudes towards human–robot interactions, whereas those high in agreeableness, extraversion, and openness are generally more receptive^8–10^. In addition, robot-related anxiety has been shown to negatively impact the quality of human–computer interactions. For instance, individuals with higher levels of anxiety toward robots display altered gaze patterns-looking less at the robot’s face and more at its hands-and exhibit reduced responsiveness during collaborative tasks^11–14^

Taken together, these findings suggest that both perceptual and psychological factors play critical roles in shaping users’ evaluative responses to avatars.

Building upon previous behavioural findings, recent neuroimaging research has begun to elucidate how variation in facial appearance influences neural processing. For instance, Mende-Siedlecki, et al., (2023) conducted a comprehensive review demonstrating that the bilateral amygdala is consistently activated in response to faces perceived as unattractive or untrustworthy. In contrast, faces rated as attractive or trustworthy elicit increased activation in regions such as the medial prefrontal cortex (mPFC), anterior cingulate cortex (ACC), medial orbitofrontal cortex (mOFC), right anterior insula, and right inferior frontal gyrus (IFG)^15^. These findings suggest that evaluations of facial trustworthiness and attractiveness engage distinct affective and social-cognitive neural circuits.

Moreover, another study indicates that avatar appearance can similarly influence brain activity during human–computer interaction. For example, in a business-oriented context involving recommendation agents, Benbasat, et al., (2020) found that mismatches in race or gender between the user and the agent elicited distinct patterns of brain activation^16^. Specifically, greater activation was observed in the right dorsolateral prefrontal cortex (DLPFC) and the dorsal ACC when participants interacted with agents whose race or gender did not match the user’s own. These activations have been interpreted as reflecting heightened cognitive conflict and emotional discomfort. Such demographic mismatches may deplete cognitive resources and impair social decision-making fluency in avatar-mediated communication. In addition to these findings, prior neuroimaging studies have shown that temporal and temporoparietal association regions are sensitive to variation in face-related and agent-related visual cues during the perception of both human faces and artificial agents. These regions have also been implicated in the integration of visual and audio-visual information during person and agent perception, including under conditions where evaluative judgments are made based on limited social input^17–23^. In the present study, these appearance-related cues, including demographic variation, were not examined as independent factors, but were treated as components of overall avatar appearance contributing to a relative selection outcome (selected vs. non-selected) during a brief first-encounter evaluation.

During the experiment, participants viewed two video clips in which avatars, differing in race, gender, or age, took turns greeting them. Participants were then asked to choose which avatars they would prefer to interact with again, without any extended dialogue or feedback. While participants watched the videos, Blood-Oxygen-Level-Dependent (BOLD) signals were recorded using fMRI. Following the scanning session, participants completed a series of impression-rating scales to evaluate each avatar’s appearance. For the analysis, neural responses were compared between avatars that were chosen for re-engagement (selected avatars) and those that were not selected (non-selected avatars).

Based on prior literature and the theoretical framework described above, the present study tested the following three hypotheses:

1. **Neural responses to non-selected avatars:** Compared to selected avatars, non-selected avatars are expected to elicit differential activation in brain regions previously implicated in evaluative and control-related processes. Based on prior neuroimaging findings in human–agent interaction, greater activity is anticipated in frontal and cingulate regions that have been associated with the processing of less preferred or cognitively demanding stimuli.
2. **Neural responses to selected avatars:** Compared to non-selected avatars, avatars that are selected for re-engagement are expected to elicit differential activation in brain regions implicated in the processing of socially relevant visual and audio-visual information. In particular, differences in activity are anticipated in response to variations in face-related and multimodal cues during person and agent perception.
3. **Modulation by individual differences:** Individual differences in personality traits, particularly openness to experiences and robot-related anxiety, are expected to modulate neural responses to selected and non-selected avatars. Specifically, variation in these traits is hypothesized to be associated with differences in the magnitude of neural activation within regions showing sensitivity to avatar selection outcomes.

## Results

A post-hoc analysis was conducted using G$*$Power^24^ to confirm the adequacy of the sample size. The analysis indicated sufficient statistical power (N = 37, Power = 0.910) for detecting medium-to-large effects. Participant characteristics are summarized in Table S1 (see Supplemental Materials). The following section presents the results of the avatar impression ratings, including comparisons of brain activity associated with selected versus non-selected avatars, and the influence of personality traits on neural responses during avatar evaluation. These analyses were conducted to explore how subjective impressions, and individual differences, modulate the neural processing of avatars differing in perceived interpersonal appeal.

### Behavioural results

#### Comparison of avatar impression between selected and non-selected avatars

**Fig. 1:**
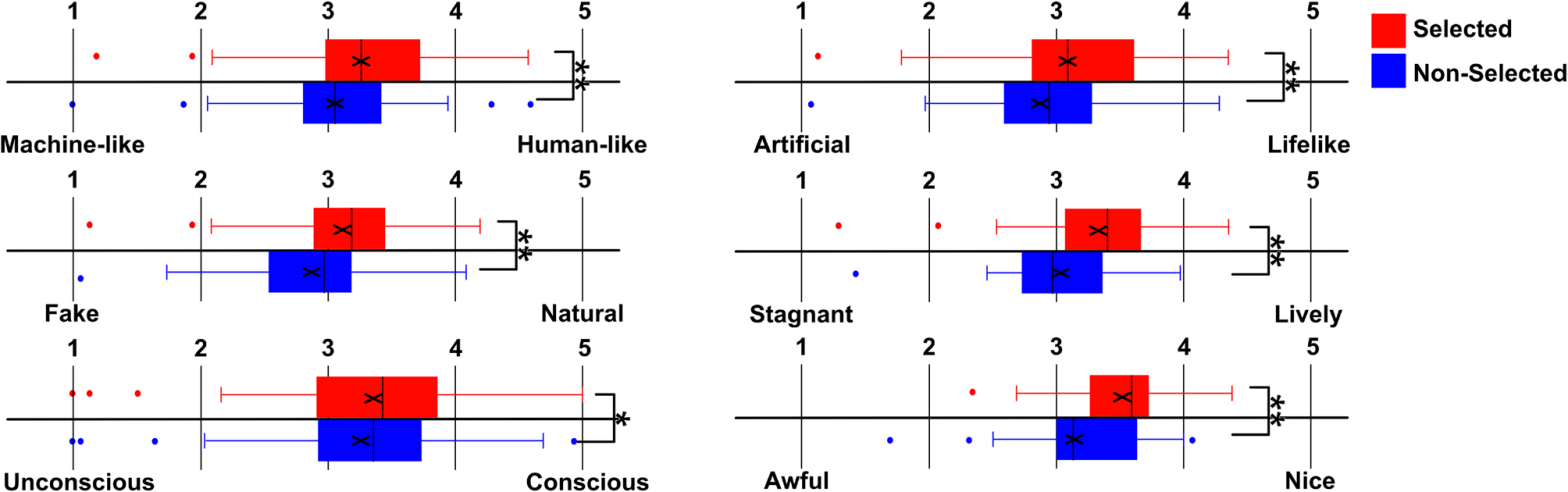
The score in avatar impressions between the selected (red boxes) and non-selected (blue boxes). The solid lines indicate the median. $*$ $*$ indicate the p-value was less than *p* < .0083 which is statistically significant after the Bonferroni correction.

**Fig. 2:**
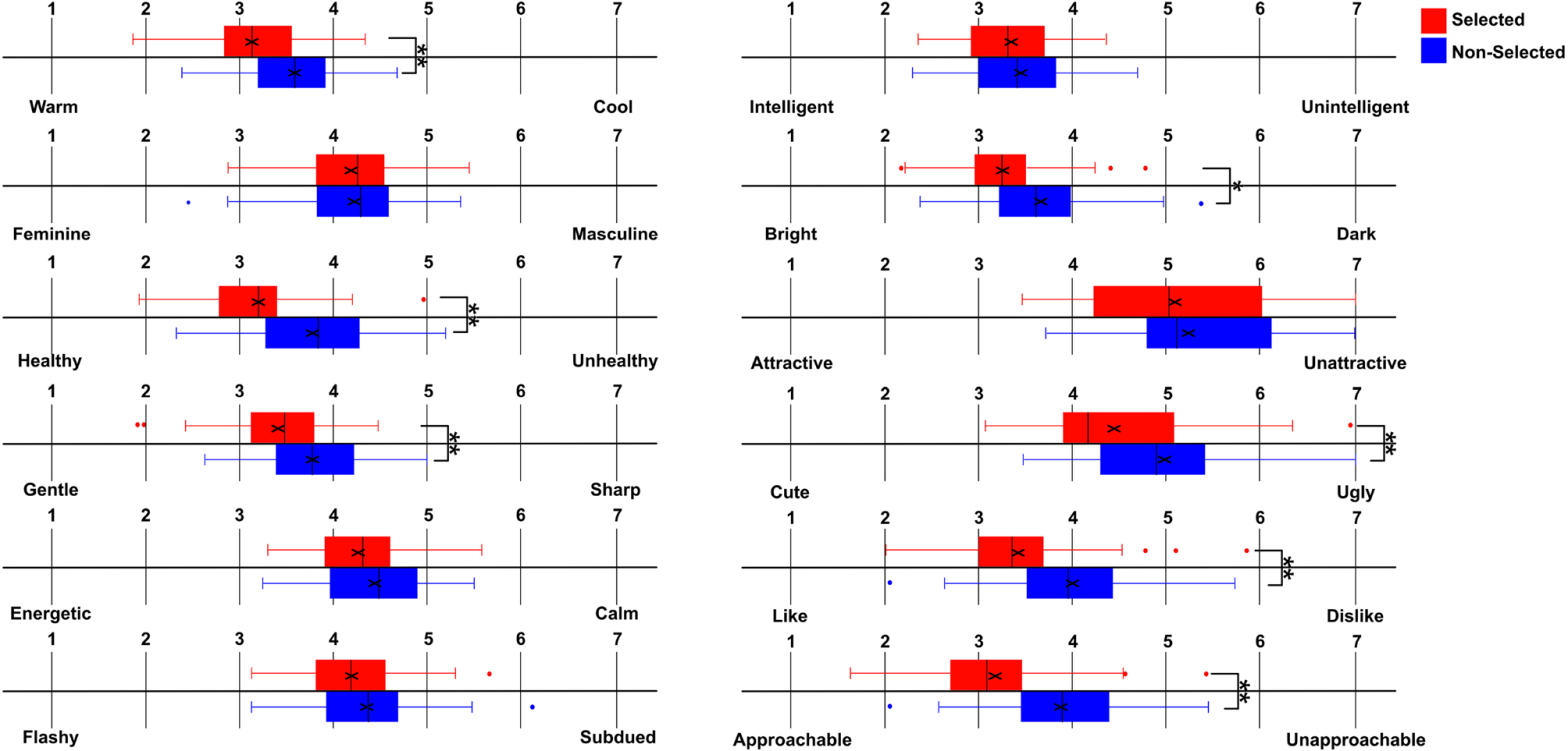
The score in facial impressions between the selected (red boxes) and non-selected (blue boxes). The solid lines indicate the median. $*$ $*$ indicate the p-value was less than *p* < .0041 which is statistically significant after the Bonferroni correction.

Figures 1 and 2 display the results of the avatar impression questionnaire, comparing selected and non-selected avatars. As shown in Fig. 1 and summarized in Table 1, significant differences were found across all impression items between selected and non-selected avatars. All comparisons reached statistical significance after applying the Bonferroni correction (adjusted *p* < .0083).

**Table 1:** Comparison of avatar impression ratings measured by the GodSpeed questionnaire between selected and non-selected avatars. For each GodSpeed item, the table reports Cohen’s *dz*, means (M), standard deviations (SD), and confidence intervals (C.I.) for selected and non-selected avatars, along with the results of paired-sample *t*-tests.

Item	Cohen’s dz	Mean(M), Standard Deviation (SD), and Confidence Interval (C.I.)	Statistical Information
Machine-like–Human-like	0.77	(Selected)M = 3.25, SD = 0.65, C.I. = 0.34 (Non-Selected)M = 3.05, SD = 0.64, C.I. = 0.34	$p = 4.15E-05, t(36) = 4.67$
Fake–Natural	0.76	(Selected)M = 3.13, SD = 0.61, C.I. = 0.32 (Non-Selected)M = 2.87, SD = 0.60, C.I. = 0.32	$p = 3.90E-05, t(36) = 4.69$
Unconscious–Conscious	0.53	(Selected)M = 3.29, SD = 0.89, C.I. = 0.47 (Non-Selected)M = 3.17, SD = 0.86, C.I. = 0.45	$p = 2.50E-03, t(36) = 3.25$
Artificial–Lifelike	0.58	(Selected)M = 3.08, SD = 0.64, C.I. = 0.34 (Non-Selected)M = 2.90, SD = 0.61, C.I. = 0.32	$p = 1.34E-03, t(36) = 3.48$
Stagnant–Lively	0.71	(Selected)M = 3.31, SD = 0.56, C.I. = 0.29 (Non-Selected)M = 3.00, SD = 0.46, C.I. = 0.24	$p = 1.50E-04, t(36) = 4.24$
Awful–Nice	0.92	(Selected)M = 3.49, SD = 0.42, C.I. = 0.22 (Non-Selected)M = 3.13, SD = 0.45, C.I. = 0.24	$p = 3.09E-06, t(36) = 5.51$

In addition, analysis of the facial impressions evaluation score revealed significant differences between the selected and non-selected avatars on several dimensions, including warm–cool, healthy–unhealthy, gentle–sharp, bright–dark, cute–ugly, like–dislike, and approachable–unapproachable (Fig. 2 and Table 2). These differences remained statistically significant after applying the Bonferroni correction (adjusted *p* < .0041). To facilitate interpretation of the magnitude of these differences, effect size (Cohen’s dz) were calculated for key comparisons and are reported in Tables 1 and 2.

**Table 2:** Comparison of facial impression rating between selected and non-selected avatars. For each facial impression item, the table reports Cohen’s *dz*, means (M), standard deviations (SD), and confidence intervals (C.I.) for selected and non-selected avatars, along with the results of paired-sample *t*-tests.

Item	Cohen’s dz	Mean(M), Standard Deviation (SD), and Confidence Interval (C.I.)	Statistical information
Warm–Cool	0.89	(Selected)M = 3.21, SD = 0.56, C.I. = 0.29 (Non-Selected)M = 3.60, SD = 0.59, C.I. = 0.31	$p = 5.22E-06, t(36) = 5.34$
Feminine–Masculine	0.002	(Selected)M = 4.18, SD = 0.52, C.I. = 0.27 (Non-Selected)M = 4.22, SD = 0.57, C.I. = 0.30	$p =.83, t(36) = -0.22$
Healthy–Unhealthy	0.84	(Selected)M = 3.18, SD = 0.62, C.I. = 0.32 (Non-Selected)M = 3.78, SD = 0.66, C.I. = 0.35	$p = 1.05E-05, t(36) = -5.12$
Gentle–Sharp	0.59	(Selected)M = 3.38, SD = 0.61, C.I. = 0.32 (Non-Selected)M = 3.76, SD = 0.63, C.I. = 0.33	$p = 8.37E-04, t(36) = -3.65$
Energetic–Calm	0.24	(Selected)M = 4.27, SD = 0.51, C.I. = 0.27 (Non-Selected)M = 4.43, SD = 0.55, C.I. = 0.29	$p =.18, t(36) = -1.38$
Flashy–Subdued	0.21	(Selected)M = 4.19, SD = 0.54, C.I. = 0.28 (Non-Selected)M = 4.35, SD = 0.64, C.I. = 0.34	$p =.24, t(36) = -1.20$
Intelligent–Unintelligent	0.20	(Selected)M = 3.35, SD = 0.50, C.I. = 0.26 (Non-Selected)M = 3.44, SD = 0.54, C.I. = 0.29	$p =.26, t(36) = -1.16$
Bright–Dark	0.57	(Selected)M = 3.26, SD = 0.51, C.I. = 0.27 (Non-Selected)M = 3.66, SD = 0.58, C.I. = 0.30	$p = 1.40E-03, t(36) = -3.46$
Attractive–Unattractive	0.42	(Selected)M = 5.15, SD = 0.99, C.I. = 0.52 (Non-Selected)M = 5.37, SD = 0.87, C.I. = 0.46	$p =.02, t(36) = -2.39$
Cute–Ugly	0.73	(Selected)M = 4.54, SD = 1.07, C.I. = 0.57 (Non-Selected)M = 4.96, SD = 0.93, C.I. = 0.49	$p = 1.79E-05, t(36) = -4.04$
Like–Dislike	1.19	(Selected)M = 3.45, SD = 0.75, C.I. = 0.39 (Non-Selected)M = 4.01, SD = 0.74, C.I. = 0.39	$p = 1.76E-08, t(36) = -7.21$
Approachable–Unapproachable	0.97	(Selected)M = 3.23, SD = 0.73, C.I. = 0.39 (Non-Selected)M = 3.88, SD = 0.72, C.I. = 0.38	$p = 9.89E-07, t(36) = -5.89$

### Neural results

#### Brain activity between selected and non-selected avatars

All whole-brain analyses were corrected for multiple comparisons at the cluster level using family-wise error (FWE) correction, with an initial voxel-level threshold of *p* < .001.

**Fig. 3:**
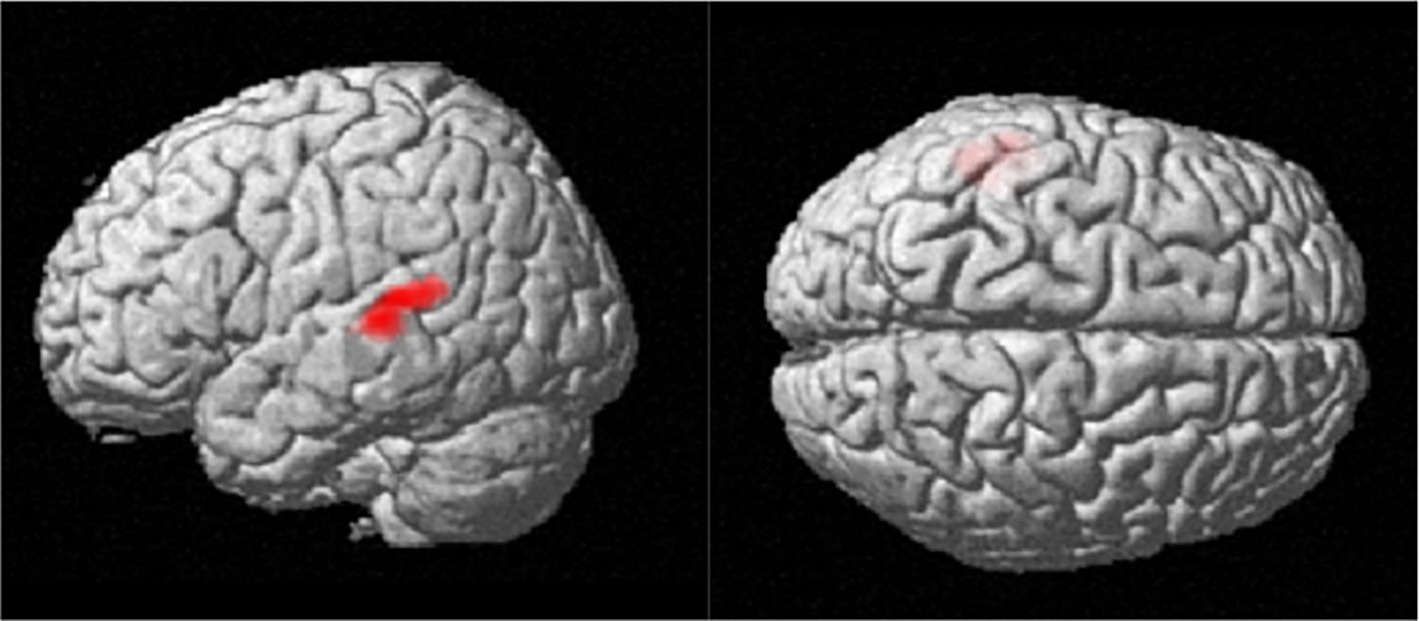
Brain regions showing greater activation for selected than non-selected avatars (voxel-level threshold *p* < .001 with the cluster-level threshold of *p* $_\textit{FWE-corrected}$<.05).

To examine neural differences between selected and non-selected avatars, blood-oxygen-level-dependent (BOLD) signals were compared during the avatar video stimulus events. Whole-brain analysis revealed significantly greater activation for selected avatars in the left middle temporal gyrus (MTG) and the left superior temporal gyrus (STG), as illustrated in Fig. 3. The cluster size, p-value, and the peak Montreal Neurological Institute (MNI) coordinates for the observed activation are summarized in Table S2 (see Supplemental Materials). No regions showed greater activation for non-selected avatars compared to selected ones.

#### Brain activity along with individual differences in selected and non-selected avatars

To examine the influence of personality traits on neural responses, BOLD signal differences between selected and non-selected avatars were analyzed in relation to the individual scores on the Ten-Item Personality Inventory (TIPI-J).

**Fig. 4:**
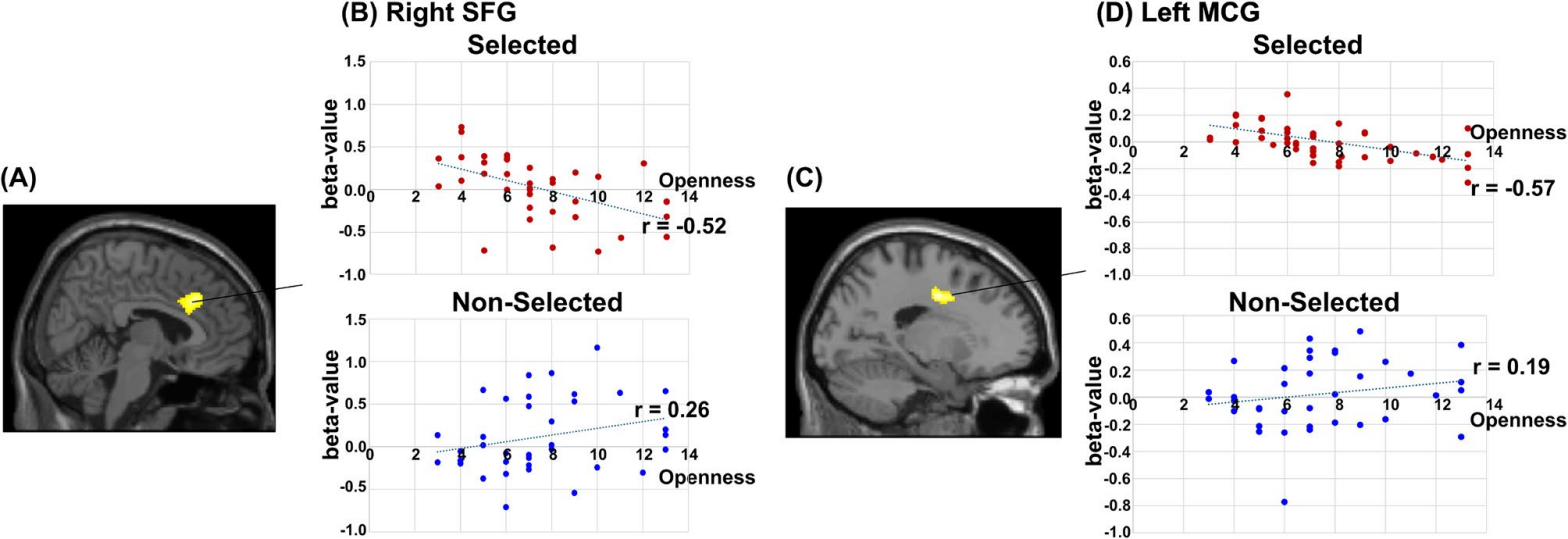
(**A**,**C**) Brian regions in which activity differences between selected and non-selected avatars were associated with the score of the openness of TIPI (voxel-level threshold *p* < .001 with the cluster-level threshold of *p* $_\textit{FWE-corrected}$<.05.). (**B**,**D**) Scatter plots showing the relationship between openness scores and beta-value in each condition. The dashed lines indicate the linear trend lines. Correlation coefficients are shown for each condition; differences between correlation strengths across conditions were not directly tested. Regression analysis on the beta-value differences (elected–non-selected) revealed significant associations with openness (SFG: *p* = .012; MCG: *p* = .036).

Figure 4A presents brain regions in which activity differences (non-selected > selected) were associated with participants’ openness scores. The cluster size, p-value, and the peak MNI coordinates for the observed activation are summarized in Table S3 (see Supplemental Materials). Specifically, in the selected avatar condition, beta values in the right superior frontal gyrus (SFG) (the peak MNI coordinates: (6,26,34); see Fig. 4A) correlated with openness scores were negatively correlated with openness scores (r = -0.52, *t(35)*< = 3.56, *p* < = .001; see upper panel of Fig. 4B). In contrast, no significant correlation was observed in the non-selected condition (r = .26, *t(35)*< = 1.60,*p* = .12; lower panel of Fig. 4B). To directly examine whether the relationship between openness and neural activity differed between conditions, an additional regression analysis was conducted on the beta-value difference between selected and non-selected avatars. This analysis revealed a significant association between openness scores and the condition-dependent beta-value difference in the SFG ($\beta$ = -0.105, t(35) = -2.65, *p* = .012). Similarly, in the selected avatar condition, beta value in the left middle cingulate gyrus (MCG)(the peak MNI coordinates: (-18, -6, 40); see Fig. 4C) were also negatively correlated with openness scores (r = -.57, *t(35)*< = 4.11, *p* < .001; upper panel of Fig. 4D), whereas no significant correlation was found in the non-selected avatar condition (r = .19, *t(35)*< = 1.16, *p* = .25; lower panel of Fig. 4D). Consistent with the findings in the SFG, regression analysis on the beta-value difference (selected–non-selected) indicated a significant association with openness in the MCG ($\beta$ = -0.044, t(35) = -2.19, *p* = .036).

## Discussion

In this section, brain activities associated with selected/non-selected and personality traits are interpreted; a conclusion is then proposed based on the three hypotheses highlighted in the Introduction.

### Brain activity towards avatars in non-selected conditions

This study aimed to investigate how differences in avatar preference influence neural responses during avatar-based interactions. The first hypothesis posited that avatars perceived as non-selected would evoke stronger evaluative or aversive responses compared to selected avatars. Contrary to this hypothesis, no brain regions exhibited significantly greater activation in response to non-selected avatars.

Accordingly, cartoon-like avatars were deliberately employed to reduce the likelihood of strong negative or uncanny responses and to focus on neural sensitivity to relative outcomes under controlled conditions. Importantly, the present experimental design was not optimized in order to elicit strong aversive responses, but rather to detect neural sensitivity to relative selection outcomes under constrained conditions. One possible explanation for this null finding relates to the overall affective valence of the avatars used. Prior research has shown that cartoon-like avatars tend to evoke more positive affective responses than highly realistic or ambiguous avatars^25^. Consistent with this, the current results from the impressions questionnaire revealed that, although non-selected avatars were rated significantly lower than selected avatars on several dimensions, their overall rating still leaned toward the positive end of the spectrum. This interpretation is consistent with prior work on avatar realism and the uncanny valley. Previous studies have shown that highly realistic or ambiguous human-like agents are more likely to elicit discomfort, avoidance, or negative affect, whereas stylized or cartoon-like representations tend to produce more stable and generally positive evaluations^26,27^. As such, the non-selected avatars may not have been sufficiently aversive to elicit robust neural differences typically associated with strongly negative or avoidance-related evaluations.

These findings suggest that, within the perceptual range employed in the present study, differences between selected and non-selected avatars may not have been large enough to engage neural systems associated with strongly negative evaluation processing. This raises important methodological considerations for future research, such as incorporating avatars with more pronounced aversive characteristics or systematically manipulating emotional expressions to amplify effective contrasts.

### Brain activity towards avatars in selected conditions

Consistent with the second hypothesis, selected avatars received higher ratings on impression measures compared to non-selected avatars. Neuroimaging data revealed increased activation in the left MTG and STG when participants viewed selected avatars. These findings align with prior research implicating the MTG in the processing of face-related and audio-visual information. For instance, Pourtois, et al. (2005) demonstrated that the MTG is responsive to congruent audio-visual stimuli^28^, and McLelland, et al. (2014) reported greater MTG activation for familiar faces compared to unfamiliar faces or objects^29^. Moreover, a previous study has reported that STG and MTG play critical roles in the integration of auditory and visual information^30^. Based on these findings, it is possible that selected avatars were associated with greater recruitment of neural processes related to audio-visual integration during the greeting videos. The observed activation patterns are consistent with the engagement of neural regions previously implicated in face-related and audio-visual processing. Recent neuroimaging studies in human–robot and human–agent interaction further support this interpretation^31,32^. For example, prior work has shown that interactions with artificial agents, compared to human partners, engage temporal and temporoparietal regions involved in social perception and the processing of communicative cues, including the STG and MTG. These regions have been associated with evaluating the social relevance and communicative intent of non-human agents, even under brief or minimal interaction conditions^31^. Other studies have similarly reported differential activation in temporal and frontal regions when participants observe or interact with robotic versus human agents, suggesting that the brain flexibly recruits social perceptual networks depending on the perceived social salience of an artificial agent^32^. However, the present data do not allow direct inferences regarding specific subjective experiences such as familiarity or emotional states. Notably, this occurred despite all avatars presenting identical verbal content and facial expressions (e.g., smiling, blinking), indicating that subtle differences in appearance or perceived social cues can significantly influence user responses.

In addition, the STG has been previously associated with sensitivity to socially relevant visual and auditory cues^33–35^. The greater STG activation observed in response to selected avatars may reflect increased sensitivity to visual and audio-visual cues conveyed by the avatars during the brief introduction phase.

### How personality traits influence neural responses in selected and non-selected conditions

Consistent with the third hypothesis, individual differences in personality traits, particularly openness to experience, modulated neural responses to selected avatars. In participants with higher openness scores (as measured by the Ten-Item Personality Inventory^36^), activity in the right SFG and left MCG was negatively correlated with avatar preference. The SFG has been implicated in self-referential processing^37,38^, decision-making^39^, and the evaluation of social relationships^40^, while the MCG is associated with value-based decision-making^41^. Openness is characterized by a desire for novelty, intellectual curiosity, and flexible thinking^42,43^. Individuals with higher openness may differ in how they approach evaluative judgments under conditions of limited information^43,44^. It is important to note that these associations are correlational in nature and do not permit causal conclusions. Accordingly, the present findings cannot determine whether openness influences neural engagement during avatar evaluation, or whether individuals with certain neurocognitive profiles tend to exhibit both higher openness and lower activation in these regions.

Overall, these findings suggest that the impression of avatars is not only shaped by surface-level visual features but also modulated by individual differences in evaluative tendencies. Understanding these associations may help inform future research on individual differences in avatar evaluation, with potential relevance for the design of user-centered avatar-based communication systems.

## Conclusion

This study has examined how individual differences, particularly personality traits, are associated with neural responses during early-stage evaluation of virtual avatars.

First, non-selected avatars did not elicit greater activation in brain regions previously associated with negative or aversive processing, suggesting that the perceptual differences between selected and non-selected avatars were relatively subtle within the stimulus set used.

Secondly, avatars selected for re-engagement were associated with increased activation in the left MTG and STG. These activations were observed in regions previously implicated in face-related and audio-visual processing, although the present data do not allow direct inferences regarding specific subjective experiences such as familiarity or emotional valence.

Thirdly, individual differences in openness to the experience were associated with variability in neural responses with frontal and cingulate regions, including the SFG and MCG. Importantly, these associations were correlational in nature and do not permit causal interpretations regarding cognitive processing strategies.

Further studies incorporating behavioural indices such as decision time, confidence ratings, or explicit strategy measures, as well as a broader range of avatar characteristics, will be necessary to clarify the cognitive and affective mechanisms underlying avatar evaluation.

## Methods

This study was approved by the Ethics Committee of Smart-Aging Research Center MRI Research Ethics Committee, Tohoku University, and Technology and study was performed in accordance with the Declaration of Helsinki (approval number:2308-01). All participants provided written informed consent.

### Participants

Forty-four healthy adults participated in the study. Demographic information including age and gender was collected. All participants provided written informed consent before the experiment, in accordance with institutional ethical guidelines. Data from two participants who displayed excessive head movement in the MRI scanner, defined as translational or rotational displacements greater than 3.0mm in any direction were excluded from this study. Therefore, the data of forty-two participants were analyzed in Whole-brain activity data(age range: 22.07± 1.88, twenty-six men and sixteen women). For the analysis of avatar impression ratings, five additional participants were excluded as a result of missing questionnaire responses.

### Personality questionnaire

#### Ten item personality inventory (TIPI-J)

The Ten Item Personality Inventory (TIPI)^36^ is widely used throughout Psychology Studies to classify and compare personality traits in many types of languages including Japanese (TIPI-J)^45^. The questionnaire consists of a ten-item scale, and each item can be scored from 1 (Disagree strongly) to 7 (Agree strongly). The five basic dimensions of personality were based on the study by Strus, et al., (2014)^46^. Furthermore, previous studies reported that TIPI-J was studied in computer technologies, such as online classes and virtual avatar and robot interactions^47,48^.

#### Measuring avatar interaction acceptance

Three questionnaires were used to measure avatar interaction acceptance, looking at technology acceptance^49^, an anxiety towards the robot scale^14^, and a negative attitude towards the robot scale^49^. These three questionnaires were validated in Japanese and are often used in virtual avatar research^50–52^. The technology acceptance questionnaire consists of fifteen questions and was rated from 1 (Disagree strongly) to 5 (Agree strongly). The negative attitudes toward the robot scale consists of fourteen items and was rated from 1 (Disagree strongly) to 5 (Agree strongly) and the anxiety towards the robot scale consists of eleven items and was rated from 1 (Very much) to 5 (Not at all).

### Avatar impression questionnaire

#### GodSpeed

The GodSpeed questionnaire is used to measure impressions towards robots^53^ or avatars^54^. The questionnaire consists of twenty-four questions, and each question is rated from 1 (Strongly disagree) to 5 (Strongly agree). It has been translated into several languages, including Japanese^55^, and Japanese GodSpeed has been validated to measure virtual avatar impressions^56^.

#### Facial impression evaluation

Ochiai et al. (2008) developed a facial impression evaluation scale in Japanese, which was published. The scale comprises twelve bipolar adjective pairs designed to capture various dimensions of facial impressions: warm–cool, feminine–masculine, healthy–unhealthy, gentle–sharp, energetic–calm, flashy–subdued, intelligent–unintelligent, bright–dark, effective–ineffective, cute–ugly, like–dislike, and approachable–unapproachable. Each item is rated on a seven-point Likert scale, where higher scores indicate stronger associations with the right-hand descriptor.

### Apparatus

Structural and functional data were obtained using a 3-Tesla MRI scanner (Philips Ingenia 3.0T; Philips, Netherlands) with a thirty-two channel head coil located at IDAC, Tohoku University. The visual stimuli were presented on an MR-compatible LCD display (BOLDscreen 32, Namoto co., Japan). The participants watched the display through a mirror attached to the head coil. The behavioural responses were collected using an MR-compatible fibre-optic button box (FUI-932, Current Designs, Philadelphia,US). The sounds were played by a noise-canceling headphone (OptoACTIVE Active Noise Control Optical MRI Communication System, Optoacoustics Ltd., Israel). The stimulus presentation and response collection were controlled using Presentation software (Version 23.0, Neurobehavioral Systems, Inc., Berkeley, CA, US).

### Stimulus

**Fig. 5:**
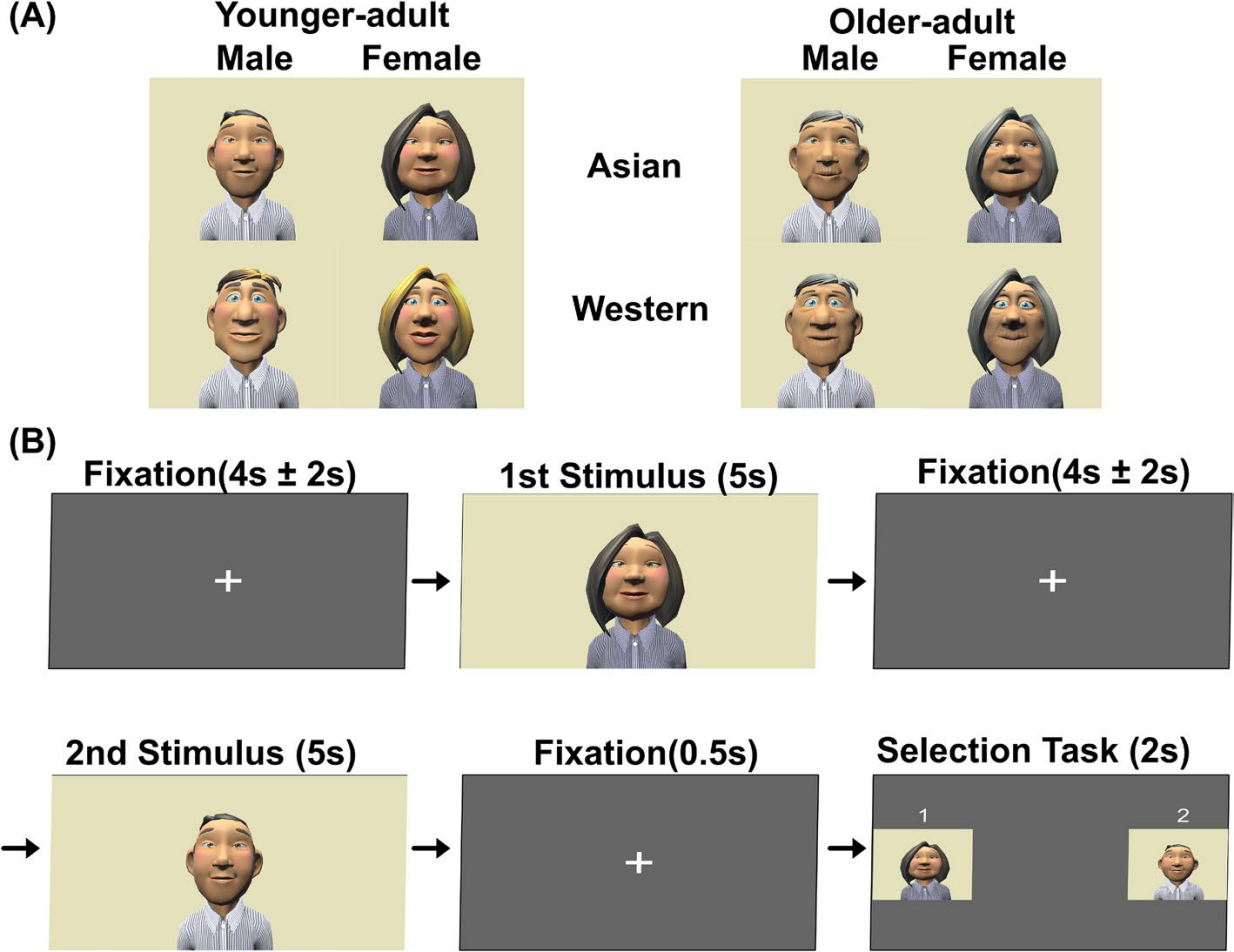
Experimental procedure in MRI scanner. Virtual avatars were created by Toon people ver 4.0 which is a Unity asset produced by JBGarraza CC BY 4.0.

Figure 5A shows example stimuli depicting the appearance of the virtual avatars used in this study. These avatars were created with three factors—genders (e.g. male and female), generations (e.g. younger- and older-adult), and race (e.g. Asian and Western). Based on prior findings by Praetorius, et al., (2021)^57^, which indicated that cartoon-style avatars are most commonly associated with contexts such as work, gaming, and social interaction. This study employed cartoon-type avatars as stimuli. To represent racial characteristics, avatars’ features were designed to resemble general Western (e.g. light skin tone, blue eyes, and blonde hair) and East Asian (e.g. light skin tone, brown eyes, and dark hair) appearance, following conventions described in previous studies^58,59^. Avatars were created using the “Toon people ver. 3.1” asset developed by JBGarraza for Unity (Unity, 2021), and included current casual clothing and hairstyle. To select avatars for the fMRI experiment, a larger pool of forty cartoon-type avatars was initially created. A separate group of nine university students, who did not participate in the fMRI experiment, evaluated these avatars prior to the main study. Avatars were presented in pairs, and for each pair, evaluators were asked to answer three forced-choice questions: (1) “Which avatar would you like to talk to?”, (2) “Which avatar do you like?”, and (3) “Which avatar would you like to be friends with?”. Based on the selection frequencies across these questions, preference distributions were calculated for each avatar. From the initial pool of forty avatars, thirty-two avatars were selected for inclusion in the fMRI experiment after excluding avatars that received consistently low preference ratings, in order to avoid extremely aversive stimuli while maintaining variability in appearance. Avatar voices were generated using the “Ondoku3” text-to-speech tool (https://ondoku3.com/ja/).

### Procedure

Figure 5B illustrates the experimental procedure. Participants completed two sessions, with a short rest period between them. Each session lasted approximately five minutes and consisted of sixteen trials, resulting in a total of thirty-two trials per participant. To mitigate potential fatigue effects, the trials were divided into two separate sessions. To control order effects, video stimuli of virtual avatars were randomly paired and presented in each trial.

Figure 5B indicates the task sequence of each trial. The first, third, and fifth phases were “Fixation” phases in which a cross was presented while the participants looked at it. The second and fourth phases were “Introduction” phases which consisted of a video, and each virtual avatar showed up and said, “Hello, nice to meet you. Let’s enjoy chatting.” in Japanese, and participants watched the greeting. It took approximately five seconds, then, the final phase showed a question in Japanese: “Which virtual avatars do you want to talk to?” (“Selection-Task” phases), and participants chose one of two avatars who had introduced themselves in the previous phases, by pressing the MRI-compatible button.

### Rating procedure

After the fMRI session, participants evaluated each avatar using a five or seven-point Likert scale. The rating items assessed perceived attributes such as likability, approachability, and overall impression. Similar impression-based evaluation procedures have been widely used in prior studies of avatar and agent perception^54^.

### fMRI experimental design and contrasts

The fMRI experiment employed an event-related design. Neural responses during the viewing of avatar greeting videos were modeled separately for avatars that were selected and those not selected for re-engagement. At the individual level, contrasts were computed between selected and non-selected avatar conditions, and these contrasts were subsequently entered into group-level analysis.

### fMRI data acquisition

Each participant’s head was held steady using foam padding to reduce head movements. Single-shot echo-planar imaging (EPI) sequences were used to acquire functional images. The EPI parameters were as follows: repetition time (TR) = 2000 milliseconds; echo time (TE) = 30 milliseconds; flip angle = 80-degrees, 32 ascending slices, thickness = 5 mm.

### fMRI activation analysis

Functional images were preprocessed using standard procedures, including slice-timing correction, realignment, normalization to the Montreal Neurological Institute (MNI) template, and spatial smoothing with a Gaussian kernel. Statistical Parametric Mapping (SPM12) software (Wellcome Department of Imaging Neuroscience, London, UK) on Matlab 2023b (MathWorks, Natick, MA, USA) was used for MRI data analysis. All coordinates are reported in MNI space. A general linear model (GLM) was used for the first-level event-related analysis of each participant. In the GLM analysis, trials of the experiment were modeled as box-car functions representing each avatar video stimuli and selection tasks onsets and duration for each condition convolved with a hemodynamic response function.

Avatar video stimuli events were modeled for a duration of five seconds from the onset time convolved with the hemodynamics response function, and the selection task event was modeled for a duration of two seconds from the onset time convolved with the hemodynamics response function. A regression model was constructed to evaluate the hemodynamic response for assessing the effects of selected or non-selected avatars in each avatar video stimuli event.

A whole-brain analysis was conducted for every contrast between conditions. A second-level analysis used contrasts from the first-level and one-sample tests to investigate group-level activation. In addition, the score of personality questionnaires was included as multiple regression to identify the effect of individual differences on brain activity in selected, and non-selected, brain activity. The initial uncorrected voxel threshold was set to *p* $<.001$. Clusters were considered as significant if they fell below a cluster-corrected *p*(FWE) = .05.

Beta values were extracted from the region of interest (ROIs) using Marsbar (http://marsbar.sourceforge.net/) toolbox for SPM^60^. These values were used to create individual-level plots representing neural activation for the contrast of interest. In addition, beta values were correlated with individual differences obtained from personality questionnaires. Two ROIs were selected for analysis based on peak activation coordinates identified in the present contrast maps: the right superior frontal gyrus (SFG) (*x = 6, y = 26, z = 34*), the left middle cingulate gyrus (MCG) (*x =18, y = -6, z = 40*). A spherical ROI with a 10-mm radius was applied for each coordinate.

## Supplementary Information

Supplementary Information.

## Data Availability

The data underlying this article will be shared on reasonable request to the corresponding author.

## References

[1] Birk, M. V. & Mandryk, R. L. Improving the efficacy of cognitive training for digital mental health interventions through avatar customization: Crowdsourced quasi-experimental study. *J. Med. Internet Res.* **21**, e10133 (2019). 30622095 10.2196/10133 PMC6329434

[2] Shin, M., Song, S. W. & Chock, T. M. Uncanny valley effects on friendship decisions in virtual social networking service. *Cyberpsychol. Behav. Soc. Netw.* **22**, 700–705 (2019). 31613640 10.1089/cyber.2019.0122

[3] Zhang, Y. G., Dang, M. Y. & Chen, H. An explorative study on the virtual world: Investigating the avatar gender and avatar age differences in their social interactions for help-seeking. *Inf. Syst. Front.* **22**, 911–925 (2020).

[4] Ya, M. G. et al. The study of ethnic attitudes during interactions with avatars in virtual environments. *Psychol. Russia State Art* **11**, 18–29 (2018).

[5] Menshikova, G. Y., Tikhomandritskaya, O. A., Saveleva, O. A. & Popova, T. V. Gender differences in interactions with avatars of diverse ethnic appearances. *Psychol. Russia State Art* **11**, 211–222 (2018).

[6] Roccas, S., Sagiv, L., Schwartz, S. H. & Knafo, A. The big five personality factors and personal values. *Pers. Soc. Psychol. Bull.* **28**, 789–801 (2002).

[7] Dunn, R. A. & Guadagno, R. E. My avatar and me-gender and personality predictors of avatar-self discrepancy. *Comput. Hum. Behav.* **28**, 97–106 (2012).

[8] Kanero, J. et al. Are tutor robots for everyone? the influence of attitudes, anxiety, and personality on robot-led language learning. *Int. J. Soc. Robot.* **14**, 297–312 (2022).

[9] Esterwood, C., Essenmacher, K., Yang, H., Zeng, F. & Robert, L. P. A meta-analysis of human personality and robot acceptance in human–robot interaction. In *Proceedings of the 2021 CHI Conference on Human Factors in Computing Systems* 1–18 (2021).

[10] Müller, S. L. & Richert, A. The big-five personality dimensions and attitudes to-wards robots: A cross sectional study. In *Proceedings of the 11th Pervasive Technologies Related to Assistive Environments Conference*, 405–408 (2018).

[11] Hinz, N.-A., Ciardo, F. & Wykowska, A. Individual differences in attitude toward robots predict behavior in human–robot interaction. In *International Conference on Social Robotics*, 64–73 (Springer, 2019).

[12] Ivaldi, S. et al. Towards engagement models that consider individual factors in HRI: On the relation of extroversion and negative attitude towards robots to gaze and speech during a human–robot assembly task: experiments with the icub humanoid. *Int. J. Soc. Robot.* **9**, 63–86 (2017).

[13] Nomura, T., Kanda, T., Suzuki, T. & Kato, K. Prediction of human behavior in human–robot interaction using psychological scales for anxiety and negative attitudes toward robots. *IEEE Trans. Rob.* **24**, 442–451 (2008).

[14] Nomura, T., Suzuki, T., Kanda, T. & Kato, K. Measurement of anxiety toward robots. In *ROMAN 2006-The 15th IEEE International Symposium on Robot and Human Interactive Communication*, 372–377 (IEEE, 2006).

[15] Mende-Siedlecki, P., Said, C. P. & Todorov, A. The social evaluation of faces: A meta-analysis of functional neuroimaging studies. *Soc. Cogn. Affect. Neurosci.* **8**, 285–299 (2013). 22287188 10.1093/scan/nsr090 PMC3594716

[16] Benbasat, I., Dimoka, A., Pavlou, P. A. & Qiu, L. The role of demographic similarity in people’s decision to interact with online anthropomorphic recommendation agents: Evidence from a functional magnetic resonance imaging (fmri) study. *Int. J. Hum Comput Stud.* **133**, 56–70 (2020).

[17] Furl, N., Garrido, L., Dolan, R. J., Driver, J. & Duchaine, B. Fusiform gyrus face selectivity relates to individual differences in facial recognition ability. *J. Cogn. Neurosci.* **23**, 1723–1740 (2011). 20617881 10.1162/jocn.2010.21545 PMC3322334

[18] Cao, R., Li, X., Brandmeir, N. J. & Wang, S. Encoding of facial features by single neurons in the human amygdala and hippocampus. *Commun. Biol.* **4**, 1394 (2021). 34907323 10.1038/s42003-021-02917-1 PMC8671411

[19] Cao, R. *et al.* Neural mechanisms of face familiarity and learning in the human amygdala and hippocampus. *Cell Rep.* **43** (2024). 10.1016/j.celrep.2023.113520 PMC10834150 38151023

[20] Choi, D., Förster, K., Alexander, N. & Kanske, P. Downsides to the empathic brain? A review of neural correlates of empathy in major depressive disorder. *Front. Hum. Neurosci.* **18**, 1456570 (2024). 39211533 10.3389/fnhum.2024.1456570 PMC11357912

[21] Acharya, S. & Shukla, S. Mirror neurons: Enigma of the metaphysical modular brain. *J. Natl. Sci. Biol. Med.* **3**, 118 (2012). 10.4103/0976-9668.101878 PMC3510904 23225972

[22] Thompson, E. L., Bird, G. & Catmur, C. Mirror neuron brain regions contribute to identifying actions, but not intentions. *Hum. Brain Mapp.* **43**, 4901–4913 (2022). 35906896 10.1002/hbm.26036 PMC9582378

[23] Wada, S. et al. Volume of the right supramarginal gyrus is associated with a maintenance of emotion recognition ability. *PLoS ONE* **16**, e0254623 (2021). 34293003 10.1371/journal.pone.0254623 PMC8297759

[24] Faul, F., Erdfelder, E., Buchner, A. & Lang, A.-G. Statistical power analyses using g* power 3.1. Tests for correlation and regression analyses *Behav. Res. Methods* **41**, 1149–1160 (2009). 10.3758/BRM.41.4.1149 19897823

[25] Yamada, Y., Kawabe, T. & Ihaya, K. Categorization difficulty is associated with negative evaluation in the “uncanny valley’’ phenomenon. *Jpn. Psychol. Res.* **55**, 20–32 (2013).

[26] Cheetham, M., Suter, P. & Jancke, L. Perceptual discrimination difficulty and familiarity in the uncanny valley: More like a “happy valley’’. *Front. Psychol.* **5**, 1219 (2014). 25477829 10.3389/fpsyg.2014.01219 PMC4237038

[27] Kätsyri, J., Förger, K., Mäkäräinen, M. & Takala, T. A review of empirical evidence on different uncanny valley hypotheses: Support for perceptual mismatch as one road to the valley of eeriness. *Front. Psychol.* **6**, 390 (2015). 25914661 10.3389/fpsyg.2015.00390 PMC4392592

[28] Pourtois, G., de Gelder, B., Bol, A. & Crommelinck, M. Perception of facial expressions and voices and of their combination in the human brain. *Cortex* **41**, 49–59 (2005). 15633706 10.1016/s0010-9452(08)70177-1

[29] McLelland, V. C., Chan, D., Ferber, S. & Barense, M. D. Stimulus familiarity modulates functional connectivity of the perirhinal cortex and anterior hippocampus during visual discrimination of faces and objects. *Front. Hum. Neurosci.* **8**, 117 (2014). 24624075 10.3389/fnhum.2014.00117 PMC3941039

[30] Beauchamp, M. S., Lee, K. E., Argall, B. D. & Martin, A. Integration of auditory and visual information about objects in superior temporal sulcus. *Neuron* **41**, 809–823 (2004). 15003179 10.1016/s0896-6273(04)00070-4

[31] Torubarova, E., Arvidsson, C., Uddén, J. & Pereira, A. Investigating conversational dynamics in human–robot interaction with FMRI. In *Proceedings of the Annual Meeting of the Cognitive Science Society*, vol. 45 (2023).

[32] Yorgancigil, E., Yildirim, F., Urgen, B. A. & Erdogan, S. B. An exploratory analysis of the neural correlates of human–robot interactions with functional near infrared spectroscopy. *Front. Hum. Neurosci.* **16**, 883905 (2022). 35923750 10.3389/fnhum.2022.883905 PMC9339604

[33] Völlm, B. A. et al. Neuronal correlates of theory of mind and empathy: A functional magnetic resonance imaging study in a nonverbal task. *Neuroimage* **29**, 90–98 (2006). 16122944 10.1016/j.neuroimage.2005.07.022

[34] Iaria, G., Fox, C. J., Waite, C. T., Aharon, I. & Barton, J. J. The contribution of the fusiform gyrus and superior temporal sulcus in processing facial attractiveness: Neuropsychological and neuroimaging evidence. *Neuroscience* **155**, 409–422 (2008). 18590800 10.1016/j.neuroscience.2008.05.046 PMC2605709

[35] Iidaka, T. Role of the fusiform gyrus and superior temporal sulcus in face perception and recognition: An empirical review. *Jpn. Psychol. Res.* **56**, 33–45 (2014).

[36] Ehrhart, M. G. et al. Testing the latent factor structure and construct validity of the ten-item personality inventory. *Personality Individ. Differ.* **47**, 900–905 (2009).

[37] Platek, S. M. et al. Neural substrates for functionally discriminating self-face from personally familiar faces. *Hum. Brain Mapp.* **27**, 91–98 (2006). 16035037 10.1002/hbm.20168 PMC6871291

[38] Platek, S. M., Keenan, J. P., Gallup, G. G. Jr. & Mohamed, F. B. Where am I? The neurological correlates of self and other. *Cogn. Brain Res.* **19**, 114–122 (2004). 10.1016/j.cogbrainres.2003.11.014 15019708

[39] Paulus, M. P., Feinstein, J. S., Leland, D. & Simmons, A. N. Superior temporal gyrus and insula provide response and outcome-dependent information during assessment and action selection in a decision-making situation. *Neuroimage* **25**, 607–615 (2005). 15784440 10.1016/j.neuroimage.2004.12.055

[40] Deen, B., Husain, G. & Freiwald, W. A. A familiar face and person processing area in the human temporal pole. *Proc. Natl. Acad. Sci.* **121**, e2321346121 (2024). 38954551 10.1073/pnas.2321346121 PMC11252731

[41] Vogt, B. A. Pain and emotion interactions in subregions of the cingulate gyrus. *Nat. Rev. Neurosci.* **6**, 533–544 (2005). 15995724 10.1038/nrn1704 PMC2659949

[42] Gong, J. et al. The relationship between openness and social anxiety: The chain mediating roles of social networking site use and self-evaluation. *BMC Psychol.* **11**, 391 (2023). 37957764 10.1186/s40359-023-01412-y PMC10644632

[43] El Othman, R., El Othman, R., Hallit, R., Obeid, S. & Hallit, S. Personality traits, emotional intelligence and decision-making styles in Lebanese universities medical students. *BMC Psychol.* **8**, 1–14 (2020). 32370782 10.1186/s40359-020-00406-4 PMC7201943

[44] Pretz, J. E. et al. Development and validation of a new measure of intuition: The types of intuition scale. *J. Behav. Decis. Mak.* **27**, 454–467 (2014).

[45] Oshio, A., Shingo, A. & Cutrone, P. Development, reliability, and validity of the japanese version of ten item personality inventory (TIPI-J). *Jpn. J. Personal./Pasonariti Kenkyu* **21** (2012).

[46] Strus, W., Cieciuch, J. & Rowiński, T. The circumplex of personality metatraits: A synthesizing model of personality based on the big five. *Rev. Gen. Psychol.* **18**, 273–286 (2014).

[47] Rivers, D. J. The role of personality traits and online academic self-efficacy in acceptance, actual use and achievement in moodle. *Educ. Inf. Technol.* **26**, 4353–4378 (2021). 10.1007/s10639-021-10478-3 PMC7923404 33679207

[48] Baba, J., Song, S., Nakanishi, J., Yoshikawa, Y. & Ishiguro, H. Local vs. avatar robot: Performance and perceived workload of service encounters in public space. *Front. Robot. AI* **8**, 778753 (2021). 10.3389/frobt.2021.778753 PMC8678513 34926593

[49] Heerink, M., Kröse, B., Evers, V. & Wielinga, B. Assessing acceptance of assistive social agent technology by older adults: The Almere model (2010).

[50] Miyamoto, T., Yamamoto, R. & Katagami, D. A comparative Japanese–US study of the acceptability of conversational lifelike agents for linguistic consideration. *J. Jpn. Soc. Fuzzy Theory Intell. Inf.* **35**, 731–735 (2023).

[51] Nomura, T. et al. What people assume about humanoid and animal-type robots: Cross-cultural analysis between Japan, Korea, and the United States. *Int. J. Humanoid Rob.* **5**, 25–46 (2008).

[52] Diana, F. et al. A cross-cultural comparison on implicit and explicit attitudes towards artificial agents. *Int. J. Soc. Robot.* **15**, 1439–1455 (2023). 37654700 10.1007/s12369-022-00917-7 PMC10465401

[53] Bartneck, C., Kulić, D., Croft, E. & Zoghbi, S. Measurement instruments for the anthropomorphism, animacy, likeability, perceived intelligence, and perceived safety of robots. *Int. J. Soc. Robot.* **1**, 71–81 (2009).

[54] Ho, C.-C. & MacDorman, K. F. Revisiting the uncanny valley theory: Developing and validating an alternative to the godspeed indices. *Comput. Hum. Behav.* **26**, 1508–1518 (2010).

[55] Bartneck, C. Godspeed questionnaire series: Translations and usage. In *International handbook of behavioral health assessment*, 1–35 (Springer, 2023).

[56] Kiuchi, K. *et al.* An exploratory study of the potential of online counseling for university students by a human-operated avatar counselor. In *Healthcare*, vol. 12, 1287 (MDPI, 2024). 10.3390/healthcare12131287 PMC11241672 38998822

[57] Praetorius, A. S., Krautmacher, L., Tullius, G. & Curio, C. User-avatar relationships in various contexts: Does context influence a users’ perception and choice of an avatar?. *Proc. Mensch und Computer* **2021**, 275–280 (2021).

[58] Peck, T. C., Good, J. J., Erickson, A., Bynum, I. & Bruder, G. Effects of transparency on perceived humanness: Implications for rendering skin tones using optical see-through displays. *IEEE Trans. Visual Comput. Graph.* **28**, 2179–2189 (2022). 10.1109/TVCG.2022.3150521 35148265

[59] Lin, B. D. et al. The genetic overlap between hair and eye color. *Twin Res. Hum. Genet.* **19**, 595–599 (2016). 27852355 10.1017/thg.2016.85

[60] Brett, M. et al. Region of interest analysis using the marsbar toolbox for SPM 99. *Neuroimage* **16**, S497 (2002).

