# Supplementary material for: Neural responses to virtual avatars are shaped by user preference and personality traits
Source: Sci Rep. 2026 Feb 10;16:8060. doi: 10.1038/s41598-026-39704-z (PMC12960805; doi:10.1038/s41598-026-39704-z)
Supplement: Supplementary file 1 — Supplementary Information. [file 41598_2026_39704_MOESM1_ESM.pdf]

Supplemental Table 1. Data of Participants

Category	Score (Average ± C.I.)
**Ten Item Personality Inventory (TIPI-J)**	
Extraversion	6.70±0.84
Conscientiousness	5.57±0.88
Agreeableness	9.05±0.60
Neuroticism	7.23±1.10
Openness	7.43±0.92
**Avatar Interaction Acceptance**	
Avatar Anxiety	2.14±0.24
Avatar Rejection	1.95±0.15
Technology Acceptance	2.42±0.17

Supplemental Table 2. Comparison of BOLD signal in Selected > Non-Selected Avatars

#Cluster	Regions	x	y	z	peak z state	cluster size	p
1	Left Planum Temporal<br>Left Superior Temporal Gyrus<br>Left Middle Temporal Gyrus<br>Left Parietal Operculum	-46	-42	12	3.96	370	0.032

Supplemental Table 3. Comparison of BOLD signal in Non-Selected > Selected Avatars along with the score of the Openness of TIPI

#Cluster	Regions	x	y	z	peak z state	cluster size	p
1	Left Middle Cinulate Gyrus<br>Left Supplementary Motor Cortex<br>Left Superior Frontal Grus<br>Left Middle Frontal Gyrus<br>Left Precentral Gyrus	-18	-6	40	5.63	351	0.030
2	Right Superior Frontal Gyrus<br>Right Middle Cingulate Gyrus<br>Right Anterior Cingualte Gyrus<br>Right Supplementary Motor Cortex<br>Left Superior Frontal Gyrus<br>Left Middle Cingulate Gyrus<br>Left Anterior Cingulate Gyrus<br>Left Supplementary Motor Cortex	6	26	34	4.81	357	0.028
